# Integrated epidemiology for vector-borne zoonoses

**DOI:** 10.1093/trstmh/trv115

**Published:** 2016-01-28

**Authors:** Nicola A. Wardrop

**Affiliations:** Geography and Environment, University of Southampton, Southampton, SO17 1BJ, UK

**Keywords:** Epidemiology, Human African trypanosomiasis, Spatial analysis, Spill-over, Vector-borne disease, Zoonoses

## Abstract

The development and application of interventions for the control of vector-borne zoonoses requires broad understanding of epidemiological linkages between vector, animal infection and human infection. However, there are significant gaps in our understanding of these linkages and a lack of appropriate data poses a considerable barrier to addressing this issue. A move towards strengthened surveillance of vectors and disease in both animal and human hosts, in combination with linked human-animal surveys, could form the backbone for epidemiological integration, enabling explicit assessment of the animal-human (and vector) interface, and subsequent implications for spill-over to human populations. Currently available data on the spatial distribution of human African trypanosomiasis allow an illustrative example.

Human health, animal health and the environment are innately connected, and in the control of zoonotic diseases, both human and animal hosts should be considered. For vector-borne zoonoses (VBZ), this extends to include vectors. The development and application of VBZ interventions requires comprehensive understanding of epidemiological linkages between vector, animal infection and human infection. Despite increasing emphasis on VBZ epidemiology at the human-animal-vector interface, significant gaps remain in our understanding of the relationships between vector, animal and human populations, and infection within these populations, in the context of disease risk.

The distribution of zoonotic pathogens in reservoir populations, and spill-over to humans, varies according to biotic, abiotic, socio-economic and behavioural factors.^1,2^ Spatial proximity and contact patterns between animals, humans and vectors play a central role in infection risk. However, co-existence of hosts and vectors does not always equate to pathogen presence, and the presence of reservoirs, vectors, humans and pathogens in a particular area is necessary for spill-over to humans but not sufficient.^3^ For many VBZ, we lack knowledge of the factors driving heterogeneity of zoonotic disease risk in animal and human populations and there is a need for greater understanding of the relationship between disease presence (or frequency) in human and animal hosts, and the influence of socio-economic, behavioural and environmental factors on heterogeneous spill-over. This hinders the implementation and targeting of VBZ control measures to protect human and animal health. For many VBZ, a lack of appropriate data prevents these issues from being adequately addressed. However, to strengthen the evidence base, available data must be utilised in an integrated manner, explicitly addressing the spatial distributions of animal hosts (and infection), human hosts (and infection) and vectors, and their inter-relationships.

Identification of areas where transmission may occur requires information on animal reservoir presence or, preferably, abundance. Spatially disaggregated estimates of livestock densities from livestock censuses or open-access modelled livestock products can provide this information for livestock reservoirs.^4^ For wildlife reservoirs it is more difficult to assess host distributions and densities. Specific surveys or species occurrence data (e.g. from the Global Biodiversity Information Facility), in combination with spatial modelling, can provide wildlife distribution estimates, although available data are generally presence only, or presence-absence, rather than abundance or density.^5^ Alternatively, proxies such as geographical extents of potential host habitats may be used. Human population distributions are available via national censuses, or spatially disaggregated products with a finer spatial resolution.^6^ The final component required is vector presence (or abundance). However, vector distribution data are not routinely available: data may not be geographically comprehensive or up-to-date, and availability depends on resources. Again spatial modelling provides the means to extrapolate available occurrence data.

Integrating information on the spatial extents of reservoir, human and vector populations enables identification of geographical areas that are potentially suitable for transmission. If the pathogen is present, animal infections will occur and the potential for spill-over to humans will exist. However, pathogen presence data can have poor availability, particularly in animal hosts. Specific surveys (preferably in linked human-animal populations) can be conducted where feasible, or information on (human) infections diagnosed by health services can be used, although comparable data in animal hosts are generally not available. Integrating these layers of data, we can identify spatial heterogeneity in epidemiological relationships and examine the drivers of transmission in animal reservoirs and spill-over to humans. Two assumptions may be that, one, transmission to humans will occur where reservoirs, vectors and humans co-exist (given pathogen presence), and, two, transmission will not occur where reservoirs, vectors and humans do not co-exist. However, challenging these assumptions and explicitly integrating epidemiological analyses of these components can deliver new insights.

The zoonotic, tsetse-transmitted disease, human African trypanosomiasis (HAT; caused by *Trypanosoma brucei rhodesiense*) presents a good example. Within south-east Uganda, where livestock are the main reservoir, we can integrate human, livestock and vector distributions^4,6,7^ to identify areas that are theoretically suitable for transmission to humans: areas where (based on available data) reservoirs, vectors and humans co-exist (Figure 1). The absence of one of these components indicates a lack of transmission potential and, thus, could be expected to equate to absence of human disease. Direct comparison with observed human infection data indicates a more complex scenario (Figure 2): several areas where reservoirs, humans and vectors co-exist did not experience (reported) HAT cases and, conversely, areas where reservoirs, vectors and humans did not overlap (i.e. not classified as suitable for transmission) have reported cases of HAT.^8^ In general, HAT occurs in specific foci within the tsetse belt (in contrast to non-human-infective animal trypanosomiasis, which is more widespread within areas of vector presence). This geo-spatial mismatch raises questions regarding the drivers of transmission among animal reservoirs and spill-over to humans. Data on parasite prevalence in reservoir hosts (not currently available) would provide significant further understanding: it is possible the parasite was not circulating in areas where no HAT was reported. Alternatively, the parasite may have been present, but while conditions necessary for spill-over were met, they were not sufficient; thus, raising the question, what contributes to sufficiency? The reporting of HAT in areas that were not classified as suitable for transmission highlights the need for further evidence to support targeted interventions. These areas predominantly represented zero catch from the tsetse survey, although this is likely to indicate low abundance (rather than vector absence), which may still sustain transmission.^9,10^ Further evidence linking vector abundance to human disease risk could enable more cost-effective resource use. Human and animal movements may also contribute to observed spatial patterns.

**Figure 1.**
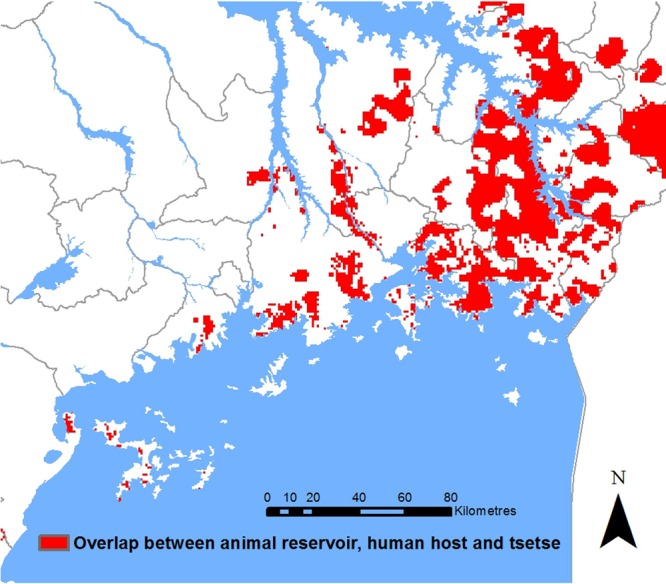
Spatial representation of theoretical suitability for *Trypanosoma brucei rhodesiense* transmission to human hosts in south-east Uganda**.** Based on spatial overlays of the distributions of livestock host populations, protected areas (as a proxy for the presence of potential wildlife hosts), tsetse populations (obtained via kernel density estimation of trap counts) and human host populations.^4,6,7^ This figure is available in black and white in print and colour at Transactions online.

**Figure 2.**
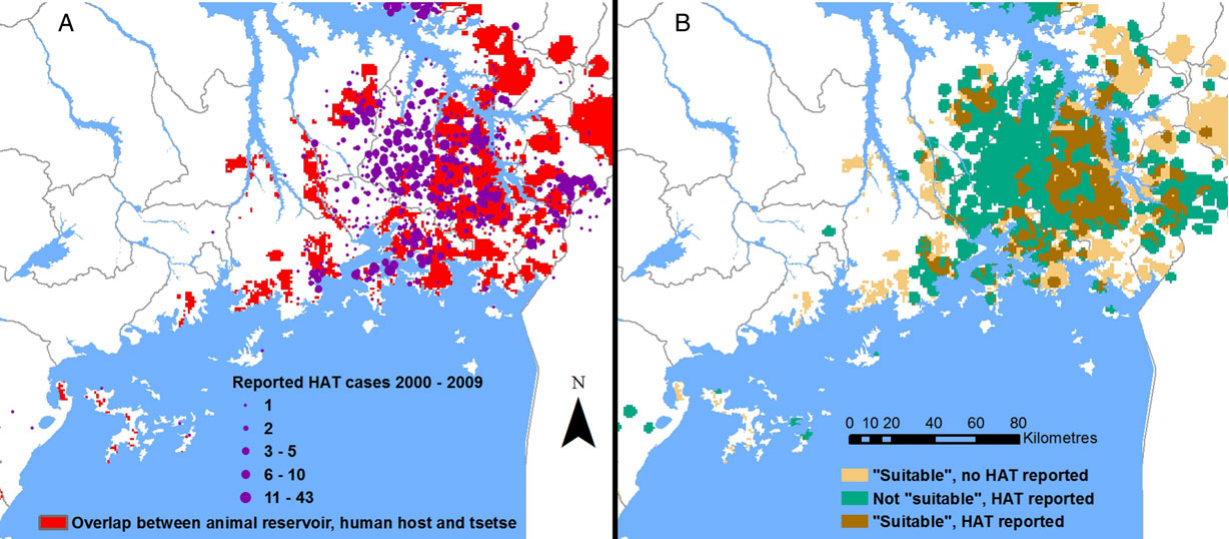
(A) Spatial overlay of theoretical suitability with reported cases of HAT from 2000 to 2009; and (B) spatial representation of concordance status (incorporating spatial smoothing around locations).^8^ This figure is available in black and white in print and colour at Transactions online.

The implementation of efficient, targeted interventions for VBZ control requires improved understanding of disease epidemiology at the human-animal-vector interface. An integrated epidemiological approach is required and strengthened surveillance of vectors, animal infection and human disease, or linked survey designs, could form the backbone for such integration. Utilising the best available data, epidemiological analyses should prioritise an integrative framework, incorporating spatial distributions of hosts and vectors, and the distributions of pathogens within these hosts; thus, explicitly addressing the animal-human-vector interface.

## References

[1] Lambin EF, Tran A, Vanwambeke SO et al Pathogenic landscapes: interactions between land, people, disease vectors, and their animal hosts. Int J Health Geogr 2010;9:54. 20979609 10.1186/1476-072X-9-54 PMC2984574

[2] Hartemink N, Vanwambeke SO, Purse BV et al Towards a resource-based habitat approach for spatial modelling of vector-borne disease risks: resource-based habitats for vector-borne diseases. Biol Rev 2015;90:1151–62. 25335785 10.1111/brv.12149

[3] Estrada-Peña A, Ostfeld RS, Peterson AT et al Effects of environmental change on zoonotic disease risk: an ecological primer. Trends Parasitol 2014;30:205–14. 24636356 10.1016/j.pt.2014.02.003

[4] Robinson TP, Wint GRW, Conchedda G et al Mapping the global distribution of livestock. PLoS One 2014;9:e96084. 24875496 10.1371/journal.pone.0096084 PMC4038494

[5] Global Biodiversity Information Facility http://www.gbif.org/ [accessed 13 October 2015].

[6] WorldPop. Worldpop alpha version 2010 estimates of numbers of people per grid square; 2013 http://www.worldpop.org.uk/ [accessed 13 October 2015].

[7] Albert M, Wardrop NA, Atkinson PM et al Tsetse Fly (*G**.* *f. fuscipes*) distribution in the Lake Victoria basin of Uganda. PLoS Negl Trop Dis 2015;9:e0003705. 25875201 10.1371/journal.pntd.0003705 PMC4398481

[8] Simarro PP, Cecchi G, Paone M et al The atlas of human African trypanosomiasis: a contribution to global mapping of neglected tropical diseases. Int J Health Geogr 2010;9:57. 21040555 10.1186/1476-072X-9-57 PMC2988709

[9] Rogers DJ Satellite imagery, tsetse and trypanosomiasis in Africa. Prev Vet Med 1991;11:201–20.

[10] Rogers DJ Trypanosomiasis ‘risk’ or ‘challenge’: a review. Acta Trop 1985;42:5–23. 2859750

